# CONUT score is associated with short-term prognosis in patients with severe acute pancreatitis: a propensity score matching cohort study

**DOI:** 10.3389/fnut.2023.1115026

**Published:** 2023-04-24

**Authors:** Lvyuan Shi, Ping Li, Lietao Wang, Dingyuan Wan, Daojin Wang, Xin Yan, Min He, Zhongwei Zhang

**Affiliations:** ^1^Department of Critical Care Medicine, West China Hospital, Sichuan University, Chengdu, Sichuan Province, China; ^2^Department of Radiology, West China Hospital, Sichuan University, Chengdu, Sichuan Province, China

**Keywords:** severe acute pancreatitis, CONUT score, short-term prognosis, mortality, ICU

## Abstract

**Background:**

The Controlling Nutritional Status (CONUT) score was designed to assess the immune-nutritional status in patients. This study aimed to investigate the role of the CONUT score in the short-term prognosis of severe acute pancreatitis.

**Methods:**

This was a retrospective cohort study. 488 patients with severe acute pancreatitis at the Department of Critical Care Medicine of the West China Hospital of Sichuan University (Chengdu, China) were enrolled in the study. Baseline data were collected from the West China Hospital of Sichuan University database. The primary outcome during follow-up was all-cause mortality. The secondary outcomes were 28 day mortality, renal insufficiency, length of stay (LOS) in the ICU, and length of stay (LOS) in the hospital. Patients were divided into two groups based on a median CONUT score of 7, and baseline differences between the two groups were eliminated by propensity matching. Univariate Cox regression analyses were performed to estimate the association between CONUT score and outcomes. The Kaplan–Meier method was used to estimate the survival rate of patients.

**Results:**

CONUT score was an independent predictor of all-cause mortality (hazard ratio [HR]:2.093; 95%CI: 1.342–3.263; *p* < 0.001) and 28 day mortality (hazard ratio [HR]:1.813; 95%CI: 1.135–2.896; *p* < 0.013). CONUT score was not statistically significant in predicting the incidence of renal insufficiency. The high CONUT group had significantly higher all-cause mortality (*p* < 0.001), and 28 day mortality (*p* < 0.011) than the low CONUT group.

**Conclusion:**

The CONUT score is an independent predictor of short-term prognosis in patients with severe acute pancreatitis, and timely nutritional support is required to reduce mortality in patients with severe acute pancreatitis.

## Introduction

Acute pancreatitis is a common clinical emergency abdomen, with a complex and variable condition that is easily treatable in mild cases and often life-threatening in heavy cases (1). The revised Atlanta classification defines acute pancreatitis with acute pancreatitis manifestations and biochemical changes, accompanied by continuous (>48 h) organ failure as severe acute pancreatitis (2). Severe acute pancreatitis has a fatality rate of up to 30%, and if it is accompanied by infection, the mortality rate will be higher (3). The current standard of care for treating severe acute pancreatitis includes an early, thorough approach with an intensive care unit (ICU) as the cornerstone, non-surgical treatment, and organ function protection as the primary emphasis (4). Among them, nutritional support therapy not only provides energy to the organism, but also prevents the evolution of the pathophysiological process of the disease, protects the barrier function of the intestinal mucosa, and is an important way to prevent infection (5–7).

However, for the assessment of a patient’s nutritional status, traditional methods include BMI, triceps skinfold thickness, and upper arm circumference (8), all of which have limitations and are susceptible to various effects such as age, gender, and race, as well as problems of measurement error. In addition, there are SGA, PG-SGA assessment forms, and NRS2002 risk screening forms (9–11), currently the NRS2002 risk screening form is commonly used in clinical practice (12). Although these assessment forms can comprehensively assess the nutritional status of patients, there are more contents to be evaluated, and the implementation is more time-consuming and energy-consuming, resulting in a decline in the execution of medical staff and non-cooperation of patients, and there are many subjective problems in the assessment content, which is easy to cause errors.

The Controlling Nutritional Status (CONUT) score, a variable based on serum albumin, total cholesterol, and total peripheral lymphocyte count (13), was originally designed to assess perioperative nutritional and immunological risk in patients undergoing gastrointestinal surgery (14). The CONUT score is easier to perform, more objective, and accurate (15). More recently, the CONUT score has also been validated for prognostic value in many other diseases (16–19). However, there are currently no studies demonstrating that CONUT scores are associated with the prognosis of severe acute pancreatitis, and we hypothesize that CONUT scores are associated with prognosis in patients with SAP. Therefore, we assessed the prognostic value of CONUT scores for short-term outcomes in patients with SAP.

We hypothesized that the CONUT score is associated with short-term prognosis in patients with severe acute pancreatitis. Therefore, we designed a retrospective cohort study to investigate the role of the CONUT score in the short-term prognosis of severe acute pancreatitis.

## Materials and methods

### Study design

The present investigation was a retrospective cohort study. All patients with severe acute pancreatitis at the Department of Critical Care Medicine of the West China Hospital of Sichuan University (Chengdu, China) from December 2015 to December 2019 were eligible for inclusion in the study. Patients younger than 10 years old, incomplete data, non-cooperation with follow-up (non-cooperation, communication difficulties, mental disorders, impaired consciousness, etc.), rescue status, chronic malnutrition and immune deficiency were excluded. Finally, we included a total of 488 severe acute pancreatitis patients in the study.

### Human subject protection

The study was approved by the Ethics Committee of the West China Hospital of Sichuan University (no: 2021-1,694), and written informed consent was obtained from all participants.

### Data collection

Data on baseline characteristics, comorbidities, and laboratory test results were collected from the West China Hospital of Sichuan University database. Clinical indicators included the patient’s surgery and infection. Laboratory variables were obtained from the results of the SAP patients’ first examination when the patients were first admitted to the ICU, including white blood cells, macrophages, lymphocytes, albumin, total cholesterol, triglycerides, serum creatinine, and total bilirubin. The CONUT score was calculated based on 3 laboratory variables: serum albumin concentration, total cholesterol concentration, and total peripheral lymphocyte count. The CONUT score was shown in Supplementary Table S1.

### Clinical outcomes

The primary outcome was all-cause mortality. The secondary outcomes were 28 day mortality, renal insufficiency, LOS in ICU, and LOS in the hospital. All-cause mortality was defined as the death of a patient due to various causes. The definition of 28 day mortality was death from various causes 28 days after admission. The definition of renal insufficiency was the 2012 version of KDIGO (20).

### Statistical analysis

Analyses were conducted using SPSS (version 25.0). All data were first checked for normality of distribution using the Kolmogorov–Smirnov test. Normally distributed data were presented as the mean ± standard deviation. Non-normally distributed data were represented as the median (inter-quartile range). Differences among the CONUT score groups were evaluated using the chi-square test for categorical variables, the *t*-test for normally distributed continuous variables, and the Mann–Whitney U test for asymmetrically distributed continuous variables. CONUT score was divided into the low CONUT (≤7) and high CONUT (>7) groups according to the median 7. The low CONUT and high CONUT groups were compared by propensity score matching (PSM). We matched each patient from the low CONUT group with a counterpart from the high CONUT group. The propensity score was the predicted probability to be in the low CONUT group, derived from a given multivariable logistic regression value of covariates. The covariates included in the propensity score calculation were age, sex, serum creatinine, and macrophages count. The matching was processed using a greedy nearest neighbor algorithm with a calliper of 0.1 times the SD of the logit of propensity score and without replacement and with random matching order. We then performed the chi-square test and Mann–Whitney U test on the matched variables, and *p* > 0.05 considered the difference between the two groups negligible. The proportional hazard assumption had to be tested before the univariate Cox regression analysis is conducted. The independent relationships between CONUT score and all-cause mortality, 28 day mortality, and renal insufficiency in the study were investigated by univariate Cox regression analyses. Univariate and multivariate COX regression analyses were performed for all-cause mortality and 28 day mortality. The Kaplan–Meier method and the log-rank test were used to estimate the survival rate of patients. A two-sided value of *p* of <0.05 was considered to indicate statistical significance.

## Results

**Figure 1:**
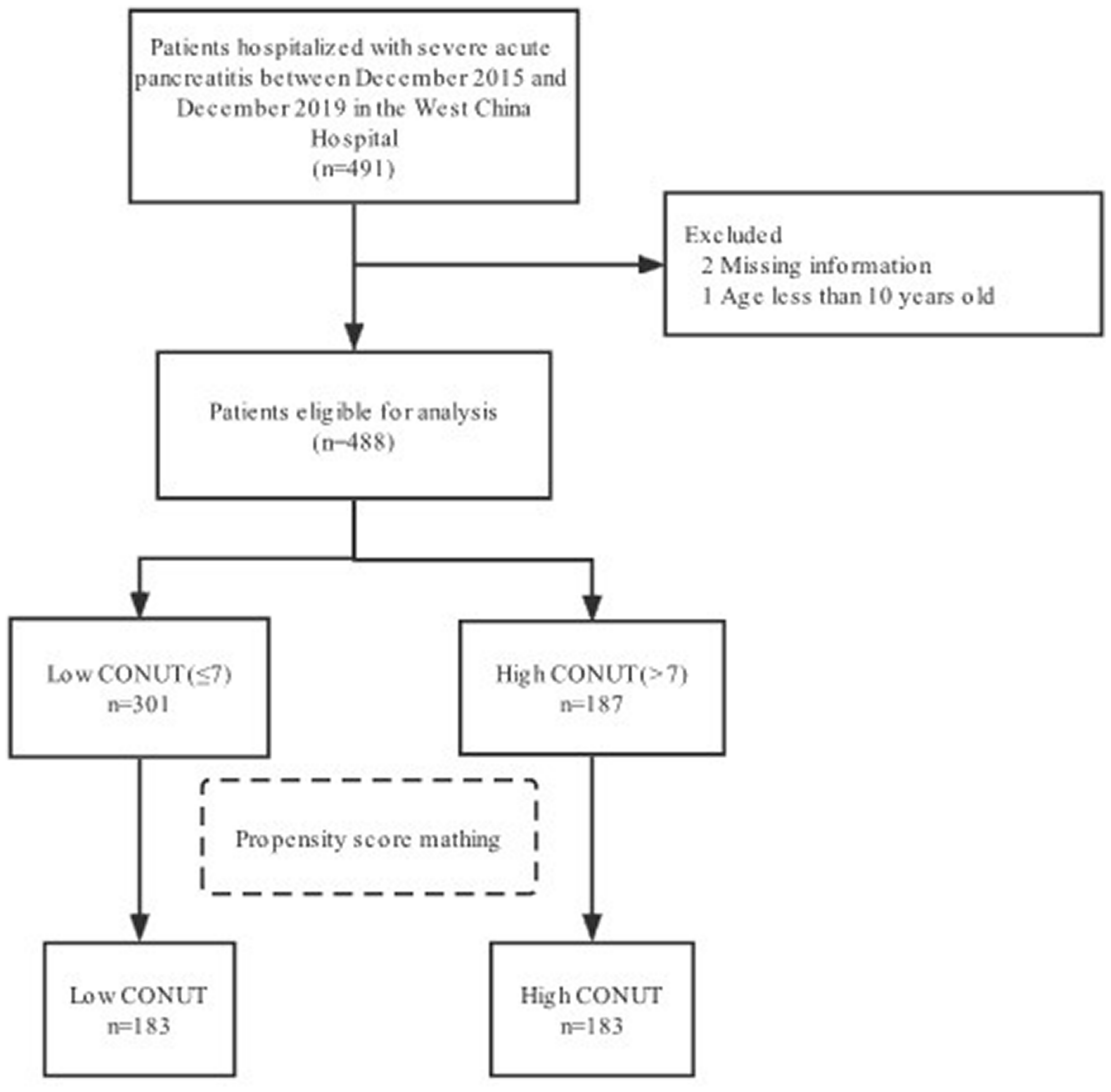
The inclusion of the study patients.

A total of 488 patients with severe acute pancreatitis who were initially admitted to the ICU between December 2015 and December 2019 (Figure 1) were categorized into the low CONUT (*n* = 301) and high CONUT (*n* = 187) groups. Table 1 presented the baseline characteristics of the study groups. The median age was 47 (37, 55) years. Most patients were men (371 cases, 65%). The median CONUT score was 7. The median of LOS in the ICU was 14.5 (7.0, 27.0) days. The median of LOS in the hospital was 24.0 (15.0, 38.0) days. During the 28 day hospital stay, 108 (22.1%) patients died. Of the outcomes at discharge, 125 (25.6%) patients died and 151 (30.9%) developed renal insufficiency. During ICU treatment, 328 (67.2%) patients underwent surgery and 318 (65.2%) patients developed co-infections. A significant difference was found in several variables between the two groups before PSM, with a two-sided *p*-value of <0.05. However, the group difference was trivial after PSM.

**Table 1:** Demographic and baseline characteristics of patients with severe acute pancreatitis.

Characteristics	Before matching	*p* value	After matching	*p* value
	All *n* = 488	Low CONUT (≤7) *n* = 301	High CONUT (>7) *n* = 187		All *n* = 366	Low CONUT *n* = 183	High CONUT *n* = 183
Ages (years)	47 (37, 55)	47.0 (36.0, 55.0)	49 (37, 55)	0.267	48.0 (37.0, 56.0)	47.0 (37.0, 58.0)	49.0 (37.0, 56.0)	0.308
Male gender (%)	317 (65%)	199 (66.1%)	118 (63.1%)	0.498	231 (63.1%)	115 (62.8%)	116 (63.4%)	0.914
All-cause mortality (%)	125 (25.6%)	63 (20.9%)	62 (33.2%)	0.003	89 (24.3%)	29 (15.8%)	60 (32.8%)	0.000
28 day mortality (%)	108 (22.1%)	57 (18.9%)	51 (27.3%)	0.031	77 (21.0%)	27 (14.8%)	50 (27.3%)	0.003
LOS in ICU (days)	14.5 (7.0,27.0)	13.0 (6.0,24.0)	16.0 (9.0,29.0)	0.006	16.0 (7.8,28.3)	15.0 (6.0,27.0)	16.0 (9.0,29.0)	0.131
LOS in hospital (days)	24.0 (15.0, 38.0)	23.0 (14.0, 38.5)	24.0 (16.0, 38.0)	0.358	25.0 (16.0, 41.3)	26.0 (15.0, 42.0)	24.0 (16.0, 38.0)	0.757
Renal insufficiency (%)	151 (30.9%)	81 (26.9%)	70 (37.4%)	0.014	123 (33.6%)	55 (30.1%)	68 (37.2%)	0.150
Surgery (%)	328 (67.2%)	203 (67.4%)	125 (66.8%)	0.891	223 (60.9%)	101 (55.2%)	122 (66.7%)	0.024
Infection (%)	318 (65.2%)	183 (60.8%)	135 (72.2%)	0.010	235 (64.2%)	103 (56.3%)	132 (72.1%)	0.002
WBC (*10^9)	12.0 (8.5, 16.8)	11.9 (8.5, 16.9)	12.1 (8.5, 16.6)	0.907	11.9 (8.3, 16.7)	11.8 (8.2, 16.7)	12.2 (8.5, 16.8)	0.740
*M* (*10^9)	0.5 (0.3, 0.7)	0.5 (0.4, 0.8)	0.5 (0.3, 0.6)	0.006	0.5 (0.3, 0.7)	0.5 (0.3, 0.7)	0.5 (0.3, 0.6)	0.270
*L* (*10^6)	975.0 (650.0, 1407.5)	1130.0 (830.0, 1665.0)	740.0 (550.0, 1010.0)	0.000	930.0 (620.0, 1337.5)	1130.0 (830.0, 1740.0)	760.0 (550.0, 1010.0)	0.000
ALB (g/dL)	3.3 (2.9, 3.8)	3.6 (3.3, 4.2)	2.9 (2.6, 3.0)	0.000	3.2 (2.8, 3.8)	3.9 (3.4, 4.4)	2.9 (2.6, 3.0)	0.000
TC (mg/dL)	3.3 (2.9, 3.8)	60.3 (44.3, 96.8)	42.6 (32.6, 62.7)	0.000	51.7 (36.0, 72.9)	61.1 (43.2, 101.3)	43.0 (32.6, 62.7)	0.000
Triglycerides (mg/dL)	2.6 (1.5, 5.3)	2.6 (1.4, 7.3)	2.6 (1.6, 4.1)	0.267	2.7 (1.5, 5.3)	2.7 (1.5, 9.3)	2.7 (1.6, 4.1)	0.125
Cr (umol/L)	89.0 (57.0, 184.0)	79.0 (57.0, 157.5)	108.0 (58.0, 240.0)	0.010	98.5 (58.0, 192.0)	83.0 (58.0, 181.0)	107.0 (58.0, 236.0)	0.203
TB (umol/L)	18.1 (11.9, 30.4)	17.6 (11.8, 28.6)	18.8 (12.1, 33.7)	0.241	18.2 (12.0, 31.7)	17.6 (12.0, 30.5)	18.8 (12.1, 33.7)	0.612

Equal proportion risk assumptions were made before COX regression, and Supplementary Figure S1 shows that the survival risk of the two curves of CONUT ≤ 7 and CONUT > 7 group changes in equal proportions, and this risk ratio does not change with time, so the PH condition of COX regression is valid. The results of after-matching groups of univariate COX regression analyses showed that the CONUT score was an independent predictor of all-cause mortality (hazard ratio [HR]: 2.093;95%CI:1.342–3.263; *p* < 0.001) and 28 day mortality(hazard ratio [HR]: 1.813;95%CI: 1.135–2.896; *p* < 0.013). CONUT score was not statistically significant in predicting the incidence of renal insufficiency (Table 2). Univariate and multivariate COX regression analyses were performed for all-cause mortality and 28 day mortality (Supplementary Tables S2, S3).

**Table 2:** Short-term complications and outcomes in the propensity score matched cohort.

Outcomes	All patients (*n* = 366)	Low CONUTS group (*n* = 183)	High CONUTS group (*n* = 183)	HR (95%CI)	*p* value
		CONUTS < 7	CONUTS ≥ 7
All-cause mortality (%)	89 (24.3%)	29 (15.8%)	60 (32.8%)	2.093 (1.342, 3.263)	0.001
28 day mortality (%)	77 (21.0%)	27 (14.8%)	50 (27.3%)	1.813 (1.135, 2.896)	0.013
Renal insufficiency (%)	123 (33.6%)	55 (30.1%)	68 (37.2%)	1.346 (0.941, 1.926)	0.104

**Figure 2:**
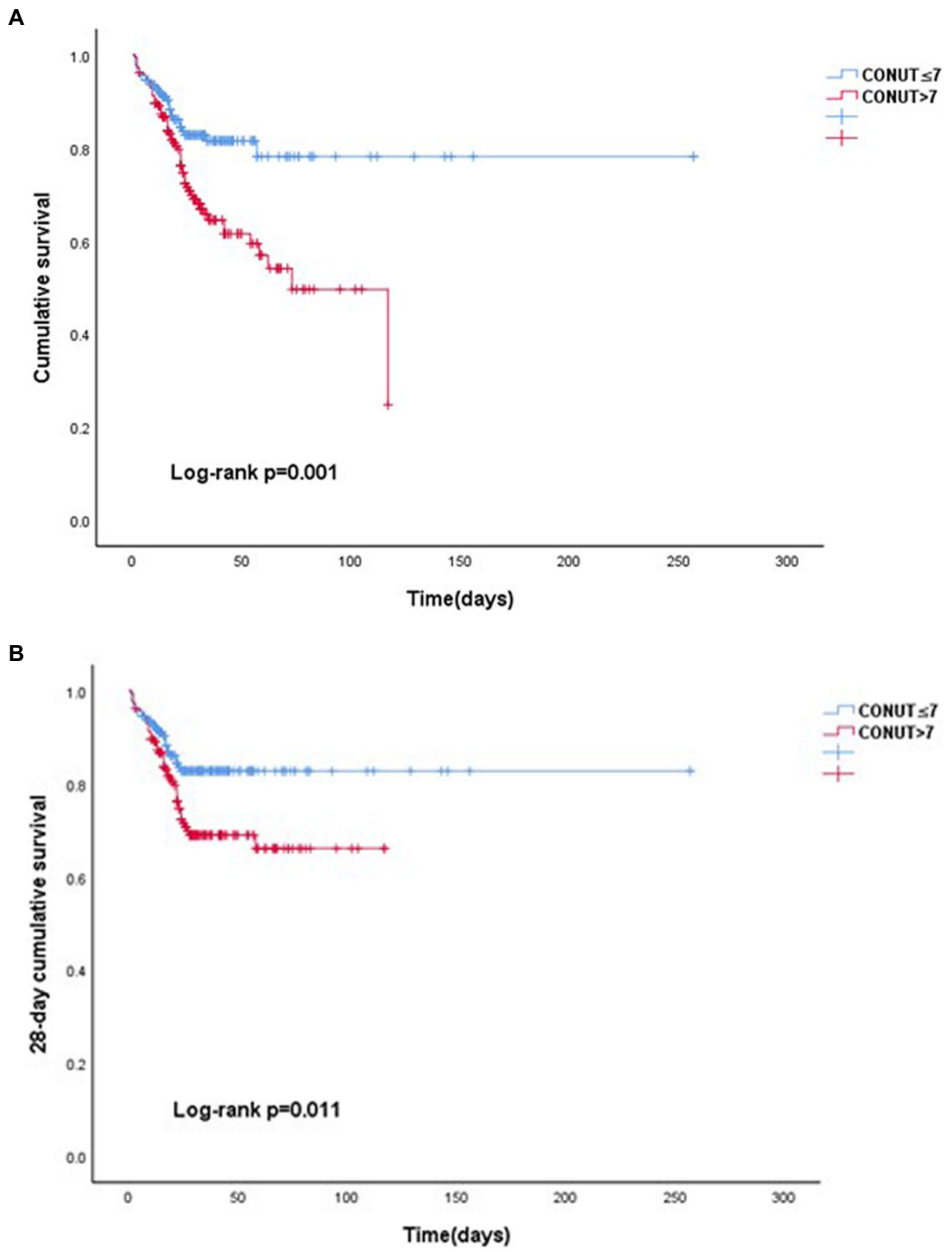
Kaplan–Meier curves for outcomes in patients with severe acute pancreatitis. **(A)**. All-cause mortality and **(B)**. 28 day mortality.

The Kaplan–Meier curve comparing the outcomes of the patients according to the median CONUT score is shown in Figures 2A,B. The high CONUT group had significantly higher all-cause mortality (*p* < 0.001), and 28 day mortality (*p* < 0.011) than the low CONUT group.

## Discussion

Mortality in severe acute pancreatitis can reach 30%, and mortality is higher if co-infected (3). There is an important relationship between nutritional status and SAP patients. Nutritional support therapy can not only provide energy for the body, but also inhibit the pathophysiological process evolution of the disease, protect the barrier function of the intestinal mucosa, and is an important way to prevent infection (5–7). To our knowledge, no studies have shown a relationship between CONUT scores and short-term outcomes in patients with acute severe pancreatitis (4). Our results suggest that the CONUT score can be used as a clinical predictor of all-cause mortality and 28 day mortality in patients with SAP. In this study, the higher the CONUT score of SAP patients when admitted to the ICU, the higher the risk of death. SAP patients have a high CONUT score (CONUT score > 7), and patients with a high CONUT score have a risk ratio of 2.093 for death compared to patients with a low CONUT score.

The Controlling Nutritional Status (CONUT) score, is a variable based on serum albumin, total cholesterol, and total peripheral lymphocyte count (13). Studies have shown that both low albumin and cholesterol are associated with in-hospital mortality in patients with SAP (21, 22). Albumin is the most abundant protein in plasma, and in the event of a disorder of nitrogen metabolism, albumin can serve as a nitrogen source to provide nutrients to tissues (23). Cholesterol is an important component of cell membranes and is also the raw material for the synthesis of many important substances in the body (24). Lymphocytes play an important role in cellular immunity (25). However, prealbumin is more sensitive to acute protein changes (26), and there is no optimal cut-off for serum albumin, cholesterol, and total lymphocyte count. In the CONUT score, the combination of these three components may better reflect the balance of immune-trophic status than univariate markers and enhance the ability to accurately predict outcomes.

The CONUT score was originally designed to assess perioperative nutritional and immunological risk in patients undergoing gastrointestinal surgery (14). In our current study, the CONUT score can also be used as an independent predictor of short-term prognosis in patients with severe acute pancreatitis, with the risk of death in the group with a high CONUT score being 2.093 times higher than the group with a low score and the 28 day risk of death being 1.913 times higher than the group with a low score. SAP patients in the group with a high CONUT score have poor nutritional status. On the one hand, SAP patients have an increased need for nutrients due to the high metabolic characteristics of the disease itself (27, 28), and mortality increases tenfold when the nitrogen balance is negative compared to patients with a positive nitrogen balance (29). On the other hand, when the patient’s nutritional status is poor, the intestinal mucosal barrier is blocked, leading to endotoxin displacement, and increasing the risk of infection (30, 31). All of this gives us reason to suspect that the CONUT score can affect the short-term prognosis of patients with SAP.

Therefore, the CONUT score can help us better monitor the nutritional status of SAP patients and prevent malnutrition and affect prognosis (15). The CONUT score can also help clinicians provide better nutritional support to patients and reduce the incidence of death in SAP patients (32, 33). For patients with severe acute pancreatitis, we should pay close attention to the nutritional status of patients, give nutritional support early, reduce the risk of malnutrition and improve the survival rate of patients (34, 35). Patients with non-severe acute pancreatitis should also be concerned about their nutritional status to prevent progression to severe acute pancreatitis (36).

On the one hand, the CONUT score evaluates the patient’s serum albumin level, total cholesterol level, and total lymphocyte count, which is simple and efficient in assessing the patient’s nutritional status and has the advantages of low cost and comprehensiveness. Moreover, it also has the advantages of objectivity and feasibility, which can be used for long-term monitoring of nutritional status, timely detection of malnutrition, and the adoption of intervention methods (32). However, on the other hand, studies have shown that CONUT, although very specific, is not very sensitive (37). And the CONUT score has its limitations because it consists of only a few laboratory indicators and lacks basic nutritional indicators, such as recent weight and appetite loss (38).

There are several limitations to this study. First, we included a small sample size in our study, and the data came from a single center in China, with a possible selection bias and a center-specific effect. Second, we did not have data on the patient’s pre-illness nutritional status, which may have existed before the disease. Also, we did not study the effect of patient overnutrition on outcomes. Finally, we did not have data on patients’ daily CONUT scores.

## Conclusion

We conclude that the CONUT score is an independent predictor of short-term prognosis in patients with severe acute pancreatitis, and timely nutritional support is required to reduce mortality in patients with severe acute pancreatitis.

## Data availability statement

The original contributions presented in the study are included in the article/Supplementary material, further inquiries can be directed to the corresponding authors.

## Ethics statement

The studies involving human participants were reviewed and approved by The ethics committee of the West China Hospital of Sichuan University (no: 2021-1,694). Written informed consent to participate in this study was provided by the participants’ legal guardian/next of kin. Written informed consent was obtained from the individual(s), and minor(s)’ legal guardian/next of kin, for the publication of any potentially identifiable images or data included in this article.

## Author contributions

LS and MH designed this study. LS carried out the study, performed statistical analyses, and drafted the article. MH and ZZ communicated with patients’ families and got their approval and critically reviewed the paper. PL, LW, DaW, XY, and DiW collected data. All authors contributed to the article and approved the submitted version.

## Funding

This research was supported by Sichuan Science and Technology Program (Project number: 2021YFS0184 and 2022YFH0002) and Project funded by China Postdoctoral Science Foundation (Project number: 2021M692298).

## Conflict of interest

The authors declare that the research was conducted in the absence of any commercial or financial relationships that could be construed as a potential conflict of interest.

## Publisher’s note

All claims expressed in this article are solely those of the authors and do not necessarily represent those of their affiliated organizations, or those of the publisher, the editors and the reviewers. Any product that may be evaluated in this article, or claim that may be made by its manufacturer, is not guaranteed or endorsed by the publisher.

## Abbreviations

CONUT: Controlling Nutritional Status
SAP: Severe Acute Pancreatitis
LOS: length of stay
ICU: Intensive Care Unit
WBC: white blood cell
M: macrophages
L: lymphocyte
ALB: albumin
TC: total cholesterol
Cr: creatinine
TB: total bilirubin
PSM: propensity score matching
