# Supplementary material for: CONUT score is associated with short-term prognosis in patients with severe acute pancreatitis: a propensity score matching cohort study
Source: Front Nutr. 2023 Apr 24;10:1115026. doi: 10.3389/fnut.2023.1115026 (PMC10165630; doi:10.3389/fnut.2023.1115026)
Supplement: Supplementary file 1 [file Data_Sheet_1.docx]

**Table S1:** Controlling nutritional status (CONUT)

| Albumin (g/dL) | >3.5 | 3.0–3.49 | 2.5–2.99 | <2.5 |
| --- | --- | --- | --- | --- |
| Score 1 | 0 | 2 | 4 | 6 |
| Total lymphocyte count  (1/μL) | >1600 | 1200–1599 | 800–1199 | <800 |
| Score 2 | 0 | 1 | 2 | 3 |
| Total cholesterol (mg/dL) | >180 | 140–179 | 100–139 | <100 |
| Score 3 | 0 | 1 | 2 | 3 |
| Nutritional impairment | Normal | Mild | Moderate | Severe |
| Score 1 + 2 + 3 | 0-1 | 2-4 | 5-8 | 9-12 |

**Table S2:** Univariate and multivariate Cox analysis of all-cause mortality in patients with severe acute pancreatitis.

| Characteristics | Univariate |  | Multivariate |  |
| --- | --- | --- | --- | --- |
|  | HR(95%CI) | P Value | HR(95%CI) | P Value |
| CONUT groups | 2.093(1.342,3.263) | 0.001 | 2.184(1.386,3.443) | 0.001 |
| Surgery | 0.900(0.585,1.386) | 0.633 | 0.840(0.540,1.305) | 0.437 |
| Infection | 0.845(0.535,1.335) | 0.470 | 0.765(0.482,1.216) | 0.257 |
| Triglycerides | 0.992(0.967,1.017) | 0.534 | 0.998(0.975,1.021) | 0.843 |

**Table S3:** Univariate and multivariate Cox analysis of 28-Day mortality in patients with severe acute pancreatitis.

| Characteristics | Univariate |  | Multivariate |  |
| --- | --- | --- | --- | --- |
|  | HR(95%CI) | P Value | HR(95%CI) | P Value |
| CONUT groups | 1.183(1.135,2.896) | 0.013 | 1.857(1.418,3.006) | 0.012 |
| Surgery | 0.776(0.493,1.221) | 0.273 | 0.718(0.453,1.138) | 0.159 |
| Infection | 0.788(0.489,1.268) | 0.326 | 0.738(0.456,1.194) | 0.215 |
| Triglycerides | 0.979(0.944,1.016) | 0.263 | 0.984(0.950,1.020) | 0.373 |

**Figure S1**: The proportional hazard assumption before the univariate Cox regression analysis

| 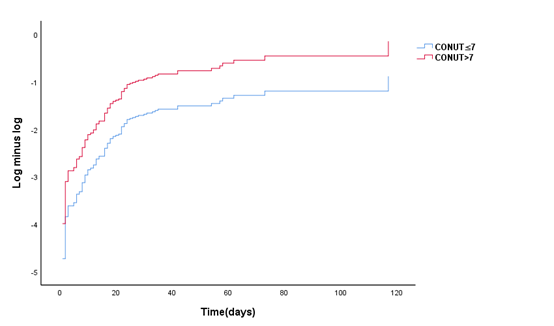 | 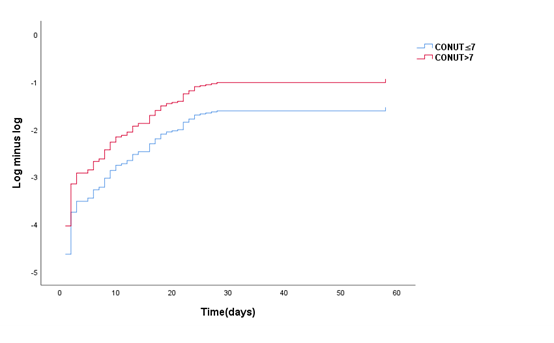 |
| --- | --- |
| a | b |
